# Microscopy of a Tooth

**Published:** 1869-12

**Authors:** S. P. Cutler

**Affiliations:** Prof. of Chemistry and Histology in the New Orleans Dental College


### MICROSCOPY OF A TOOTH.

BY S. P. CUTLER, M. D., A. E., D. D. S.
Prof. of Chemistry and Histology in the New Orleans Dental College.

A few days since I extracted a left lower molar tooth for a girl fourteen years of age. This tooth had been filled some three years ago, though not well. When I examined the remaining, though extensively decayed on all sides, except one, with an exposure of pulp and in an aching condition had been troublesome for some time.

On extracting the tooth, I as usual made an examination into its sanctum sanctorum to see what I could discover. I

first split open the tooth by driving a cutting instrument between the roots, and, as I expected, found apparently a normal pulp which was torn in too by the splitting of the tooth, and on further examination I found a nodulosa in about the center of the pulp, and on extracting the pulp from each root, which required unusual force, and on further examination of the rent pulp I found a long needle, shaped or aciculated spicula down in the root portion of each pulp, about the middle portion. I made a microscopic specimen, first of the soft pulp, with about the usual results, with an additional phenomenon, which I had long been in search of in vain, and now I may exclaim, *Eureka*; that is to say on the surface of the pulp membrane of one of the roots, to some extent, I discovered true conoidal epithelia projecting boldly up from the surface, about as close together or as far apart as the tubuli at pulp cavity.

I could not be mistaken in this case, as it was something I had long been in search of, but had never before clearly and distinctly recognized any thing of the kind, still I had entertained such ideas from analogy as all known surfaces of membranes are known to be covered with epithelia of some form or other. These epithelia I suppose, project up between the nerve fibrillar openings, and may be the true factors (if I may be allowed such an expression).

I had formerly conceived the idea that this was an exudation through these openings that I have often mentioned in former publications, but now there is *tertium quid* before us to deal with, and who shall decide. Only one portion of this pulp showed epithelia distinctly. I had almost lost hope of finding any, and was not in this case in search of any, hence it must be looked upon more as an accident than any sagacity on my part, but I hear Mr. Smith asking over the way why don't you give us your process of preparing your specimens, so that we can make the same discoveries, or so as to recognize yours? I simply reply to our friend that I scuttled the pulp by removal of the spicula or nodules, then I placed the battered fragments between two slips of glass, with a little warm pitch, then placed it under the instrument, and you know the rest.

I will state here that I always have slips of glass in readiness, lamp and pitch in my office, and I make it an invariable rule to split open all teeth I extract, that have nerves in them, and at once place between the slips of glass, and in five minutes, even if in a hurry, and the specimen is ready for examination, which I generally make at night, and take notes, the results of which have been given to the profession. After a man has accustomed himself to this plan it is very little trouble, and will repay him for his time, so it appears to me. This tooth I did not attempt to save at all by my process, as I seldom destroy tooth pulps in subjects so young, unless it be in the six-year molars, for this reason, in young subjects we never absolutely know at what precise time the apical forumin is perfected by dentification and cementification. In this case the openings were large open funnel shaped, not closed at all; the tooth probably had been erupted four or five years. I published an article on this subject in the DENTAL REGISTER, during the winter of 1867, while at Cincinnati, based upon observations made of the specimens in the Ohio Dental College. In a practical point of view what would have been the result of divitalization of pulp in the above named case, and an attempt to fill the roots; undoubtedly a failure, as the apical openings are larger than those of the canal up the root, and in all probability had this tooth remained sound, several years must have elapsed before the process would have been complete. This I regard as an interesting case worthy of attention.

In all the published articles I have seen I have never observed anything on the subject of the open condition of roots in young subjects, in relation to killing pulps, which I think at least worth the consideration of the profession. We know, as a general thing, that the tooth is fully grown before this closure takes place, and that this is the last closing out of the primary series of tooth growth, sometimes being completed soon after the full eruption, and sometimes several years elapse, as in the above cited case. The destruction of pulps in such teeth would be attended with more than ordinary difficulty, and after devitalization the removal, where there are no nodules, would be usually difficult, as in the above named case, even after the tooth's removal; in fact I believe the entire removal by broaches or any other method almost impossible, owing to the large amount of tissue and funnel shaped opening of root. Even admitting the full practicability of entire removal, another almost insurmountable difficulty presents itself that is the excess of tissue at the open root, which must of necessity continue some distance from the point of the root into the Dental socket, in consequence furnishing space and tissue for diseased action and formation of abscess with more than ordinary facility. The more ready absorption of the bone at this age furnishes another favorable circumstance for the enlargement of sack, besides other constitutional tendencies that might be named. I am treating an upper bicuspid in the same mouth, so far successfully by destroying the pulp. I had filled the tooth, approximal posterior cavity a few days ago, and on excavating there was a very small exposure of pulp, which I slightly touched with a very delicate excavator, not enough to cause more than momentary pain and no blood, the cavity small. After a day or two the tooth commenced to be sore and tender, increasing for several days, when she returned for the purpose of having it removed. I with some difficulty removed the filling, and treated the usual way, with, so far, entire success, the tooth entirely dead and relieved, and I am confident of saving it yet, though not if the foramin is open and funnel shaped like the other tooth.

